# Leadership Development Among Senior Nursing Students: A Mixed-Method Study

**DOI:** 10.7759/cureus.108679

**Published:** 2026-05-11

**Authors:** Saraswathy Kannappan, Sharadhaa Sathiyanarayanan, Vijayalakshmi K, Nesa Sathya Satchi, Vijayalakshmi Ramanujam

**Affiliations:** 1 Obstetrics and Gynecology Nursing, Apollo College of Nursing, The Tamil Nadu Dr. M.G.R. Medical University, Chennai, IND; 2 Coronary Care Unit, Apollo Hospitals, Chennai, IND; 3 Mental Health Nursing, Apollo College of Nursing, The Tamil Nadu Dr. M.G.R. Medical University, Chennai, IND; 4 Child Health Nursing, Apollo College of Nursing, The Tamil Nadu Dr. M.G.R. Medical University, Chennai, IND; 5 Obstetrics and Gynecology Nursing, Saveetha College of Nursing, Saveetha Institute of Medical and Technical Sciences, Chennai, IND

**Keywords:** challenges, clinical leadership, leadership development, leadership opportunities, senior students, student mentorship

## Abstract

Introduction: The development of leadership skills is an essential aspect of nursing education; however, their effective implementation and influence among senior nursing students have not been thoroughly examined. This research investigates leadership styles and the opportunities and challenges associated with leadership development. Materials and methods: This mixed-methods research employed a total enumerative sampling technique, a type of non-probability sampling, involving 200 of all third- and fourth-year B.Sc. Nursing students from the selected College of Nursing in Chennai. Quantitative data were gathered using the Blake and Mouton managerial grid leadership self-assessment tool, along with a Likert-scale rating to evaluate perceived opportunities and challenges in leadership development (Cronbach's α = 0.81 and 0.79, respectively). Qualitative data were collected through semi-structured interviews with 10 randomly selected participants. Statistical analysis included descriptive statistics, independent samples t-tests, and effect size calculations (Cohen’s d). Qualitative data were analyzed thematically using Braun and Clarke’s framework. Results: Team-oriented leadership was predominant (third year: 98%; fourth year: 93%). Statistically significant differences were observed between third- and fourth-year students in people-oriented (mean difference = 2.4, t = 2.51, p = 0.01, Cohen’s d = 0.35), task-oriented (mean difference = 2.64, t = 2.83, p = 0.004, Cohen’s d = 0.40), and global leadership scores (mean difference = 5.04, t = 2.71, p = 0.007, Cohen’s d = 0.38). Fourth-year students reported greater opportunities in clinical and simulation domains, while third-years highlighted mentorship support. Challenges included workload (64%), socio-cultural barriers (62%), and limited institutional support (60%). Qualitative findings reinforced the need for practical exposure, inclusive leadership roles, and faculty mentorship. Conclusions: Senior nursing students demonstrated predominantly participatory leadership styles, with significant differences across academic levels. While opportunities increased with seniority, persistent challenges limited leadership development. Structured leadership training, mentorship, and experiential learning are recommended to strengthen leadership readiness.

## Introduction

Leadership in nursing is not merely a professional competency but a transformative force that shapes patient outcomes, team dynamics, and the future of healthcare systems. Despite their recognized importance, undergraduate nursing curricula often prioritize clinical and technical skills over structured leadership development, leaving a gap in students' preparation for complex healthcare environments [1].

Recent scholarship underscores the urgency of addressing this gap. Costa et al. argue that leadership must be scaffolded progressively throughout undergraduate education to build professional identity and readiness for clinical practice [2]. Abdul-Rahim et al. highlight mentorship, communication, and reflective practice as critical enablers, while Mohammadpourhodki et al. demonstrate the potential of peer-led initiatives such as student conferences and supervision models [3,4]. However, these approaches often assume universal applicability and overlook contextual differences across healthcare systems.

Transformational leadership, frequently celebrated for fostering motivation and innovation, has shown consistent positive associations with job satisfaction and productivity across diverse settings. Yet, systematic reviews reveal variability in effect sizes, with stronger outcomes in hierarchical healthcare systems [5]. This suggests that leadership outcomes are not inherent to style alone but mediated by organizational culture and structural conditions. At the same time, barriers such as workload, socio-cultural expectations, and limited institutional support continue to hinder leadership development, particularly in resource-constrained environments [6].

Taken together, the literature highlights both promise and limitations. While participatory and transformational models are widely endorsed, their effectiveness is context-dependent, shaped by mentorship, institutional culture, and systemic constraints. This gap underscores the need for context-sensitive research that moves beyond descriptive accounts to critically evaluate how leadership competencies can be cultivated and sustained in diverse nursing education environments.

Against this backdrop, the present study investigates leadership styles, opportunities, and challenges among senior nursing students in Chennai. By employing a mixed-methods design, it seeks to provide both measurable insights and nuanced perspectives, thereby contributing to the growing body of evidence advocating for leadership-focused nursing education.

This mixed-method study aims to examine the leadership styles, opportunities, and challenges in leadership development among senior B.Sc. Nursing students at a selected college in Chennai, Tamil Nadu. Specifically, the study seeks to assess the leadership styles demonstrated by senior nursing students and examine their perceived opportunities and challenges in leadership development. It also aims to compare the leadership styles between III- and IV-year B.Sc. nursing students and explore the factors influencing leadership development among senior nursing students. The study is guided by the hypothesis that there will be a significant difference in leadership styles between III- and IV-year nursing students at the p < 0.05 level.

## Materials and methods

Research design

The study adopted a mixed-methods research design, combining quantitative and qualitative approaches to assess and explore leadership styles, opportunities, and challenges among senior nursing students. The mixed-method design was chosen to obtain both measurable data and deeper insights into the students’ leadership experiences. The study was conducted at the selected College of Nursing, Chennai, Tamil Nadu, a reputed institution known for its high academic standards and extensive clinical exposure. The study population included senior B.Sc. Nursing students from the third and fourth years. A total enumerative sampling technique was used for the quantitative strand, which included 200 participants, 100 from the third year and 100 from the fourth year, who met the inclusion criteria. For the qualitative strand, 10 participants were randomly selected from the same population to capture diverse perspectives and experiences related to leadership development. Sample size was determined using the formula (ZSD/d)² based on the pilot study findings (40.5 ± 8). Hence, the required sample size was 96 per batch, which was rounded up to 100. Thus, the total sample size was 200. The inclusion criteria comprised students enrolled in the third and fourth years of the B.Sc. Nursing programs that were available and willing to participate after providing informed consent. Students who were absent during data collection or did not consent to participate were excluded.

Materials

The study utilized a combination of standardized and self-structured instruments to ensure comprehensive data collection. A structured proforma for background variables was used to collect demographic and academic details, including age, year of study, prior leadership roles, and exposure to leadership training. Leadership style was assessed using the Blake and Mouton Managerial Grid Leadership Self-Assessment Questionnaire [7], a standardized instrument comprising 18 items equally distributed across task-oriented and people-oriented domains, rated on a five-point Likert scale. The tool has established reliability in previous studies; in the present study, internal consistency was confirmed with a Cronbach’s α of 0.82, indicating good reliability.

Additionally, two self-structured scales were developed by the researchers to assess leadership opportunities and challenges among nursing students. Each scale consisted of 12 items measured on a five-point Likert scale. Content validity of these instruments was established through expert evaluation by five specialists in nursing education and management, yielding a Content Validity Index of 0.89. The tools were pilot-tested among 20 students to ensure clarity and feasibility. Internal consistency reliability was acceptable, with Cronbach’s α values of 0.81 for the leadership opportunities scale and 0.79 for the leadership challenges scale.

For the qualitative component, a semi-structured interview guide was developed to explore students’ perceptions of leadership, opportunities for leadership development, challenges encountered, and suggestions for improvement. Subject experts reviewed the guide to ensure content relevance and clarity, thereby enhancing its validity.

Ethical Considerations

Ethical approval was obtained from the Institutional Ethics Committee of Apollo College of Nursing under IEC No. ACONC/IEC/2025/14. Before data collection, informed and written consent was obtained from all participants, who were assured of confidentiality, anonymity, and the right to withdraw from the study at any stage without repercussions.

Data collection

Data collection was carried out in two phases to align with the mixed-method design. In the quantitative phase, data were collected using a structured online questionnaire administered through Google Forms (Google LLC, Mountain View, CA, USA), which included the background variables proforma, the standardized leadership style assessment tool, and the self-structured scales on leadership opportunities and challenges. Measures were implemented to ensure data quality, including mandatory response fields to minimize missing data, restriction of duplicate entries, and systematic screening of responses for completeness, consistency, and accuracy prior to analysis.

In the qualitative phase, in-depth, semi-structured interviews were conducted with selected participants in a private, comfortable setting to facilitate open, honest responses. Each interview lasted approximately 30-40 minutes and was audio-recorded with prior informed consent. The recordings were transcribed verbatim, and the transcripts were cross-verified against the original audio files to ensure the accuracy and completeness of the data.

## Results

Quantitative data were analyzed using SPSS Statistics version 21.0 (IBM Corp. Released 2012. IBM SPSS Statistics for Windows, Version 21.0. Armonk, NY: IBM Corp.). Descriptive statistics, including frequencies, percentages, means, and standard deviations, were used to summarize demographic characteristics and study variables. The independent-samples t-test was used to assess differences in leadership styles between third- and fourth-year nursing students. Statistical significance was set at p < 0.05. In addition to p-values, effect sizes were calculated using Cohen’s d to quantify the magnitude of differences between groups, with values interpreted as small (0.2), medium (0.5), and large (0.8).

Qualitative data were analyzed using thematic analysis based on the Braun and Clarke framework. The process involved familiarization with the data through repeated readings of transcripts, the generation of initial codes, the categorization of codes, and the development of themes and subthemes that reflect participants’ experiences and perceptions of leadership, opportunities, and challenges. Themes were reviewed and refined to ensure coherence and alignment with study objectives. Methodological rigor was ensured through member checking, maintenance of an audit trail, and independent coding by two researchers to enhance credibility, dependability, and confirmability. Verbatim quotations were used to support the findings and enhance the authenticity of the qualitative results.

Table 1 shows that equal numbers of students (50%) participated from the third and fourth years, most students (70%) were aged 21 or younger, and 71.5% had served in leadership roles, whereas only 20% had received leadership training.

**Table 1:** Frequency and percentage distribution of background variables among senior nursing students (N = 200) SNA: student nurses' association, NSS: national service scheme

Background variables	f	%
Year of study		
Third year	100	50
Fourth year	100	50
Age		
Up to 21 years	140	70
Above 21 years	60	30
Leadership position in college		
Nil	57	28.5
SNA representative	20	10
Class representative	101	50.5
Red ribbon club representative	1	0.5
Youth Red Cross representative	-	-
NSS representative	3	1.5
More than 1 leadership position	18	9
Leadership training		
Yes	40	20
No	160	80

**Figure 1:**
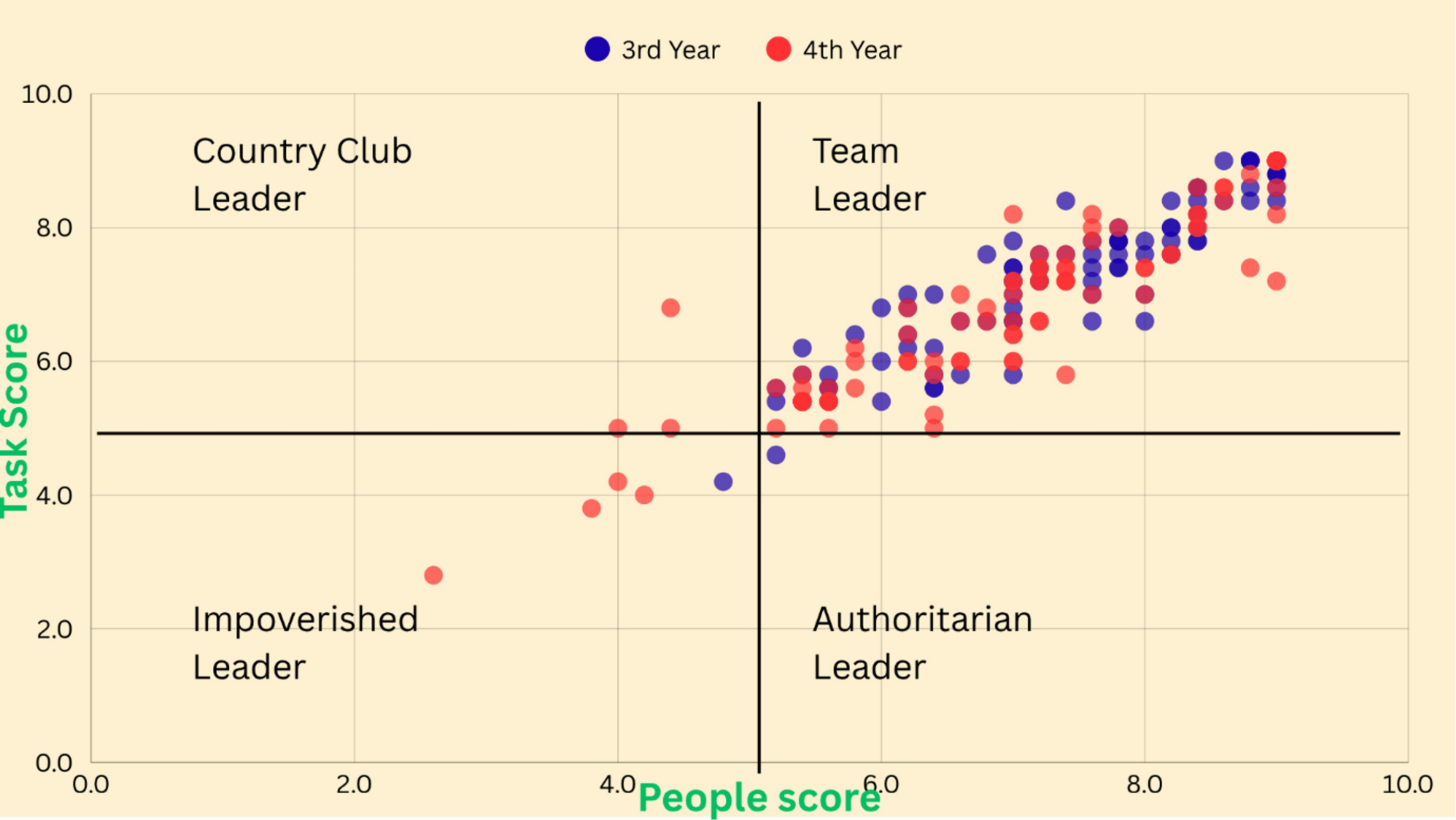
Percentage distribution of leadership styles among senior nursing students

Figure 1 illustrates that most students (98% and 93%) exhibited a team-leadership style in the third and fourth years, respectively. Very few exhibited authoritarian, country-club, or impoverished leadership styles.

**Figure 2:**
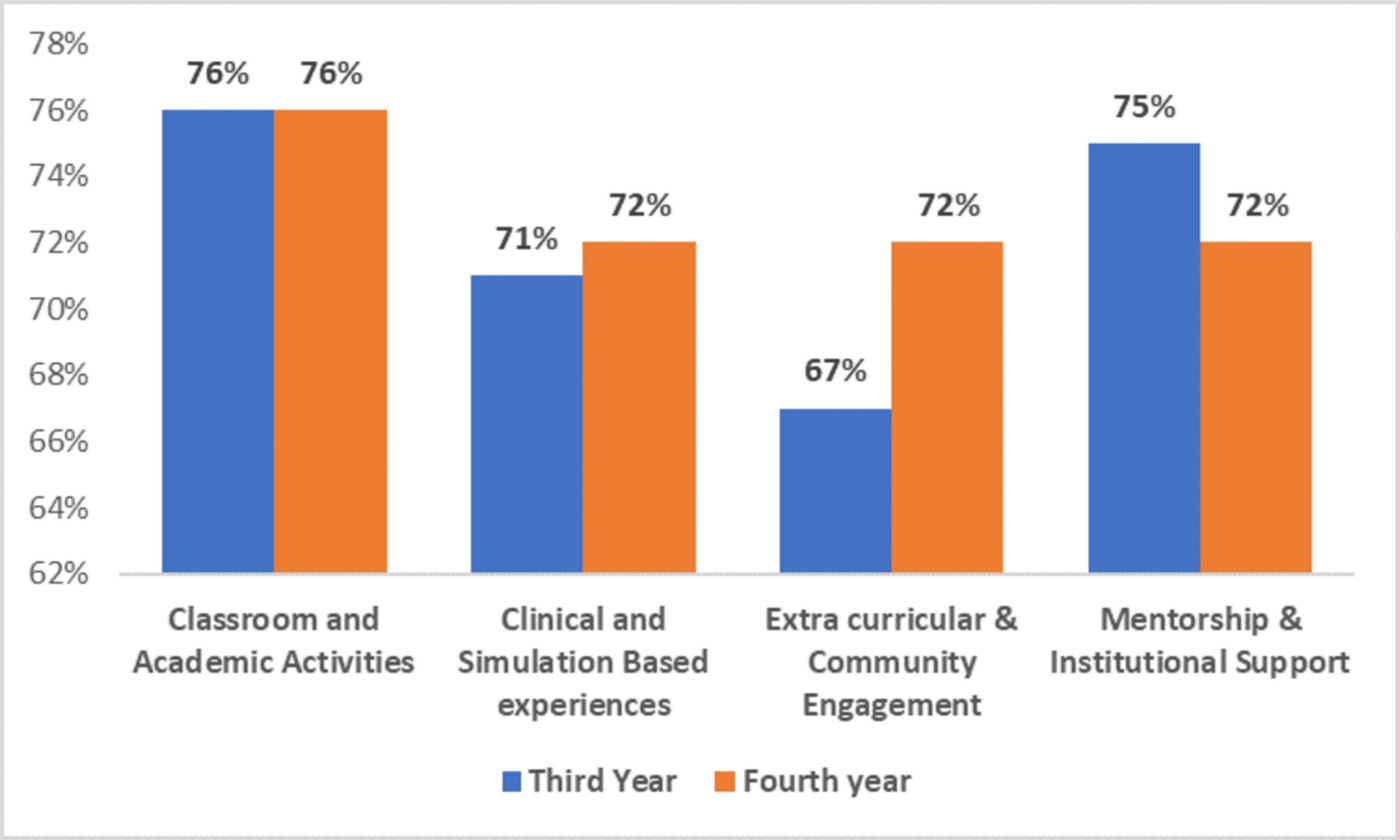
Percentage distribution of leadership opportunities among senior nursing students

Figure 2 shows that both years showed equal opportunities in the classroom and academic activities (76%). Fourth-year students had slightly higher opportunities in clinical cum simulation and extracurricular and community affairs, while third-year students reported better mentorship and institutional support (75%).

**Figure 3:**
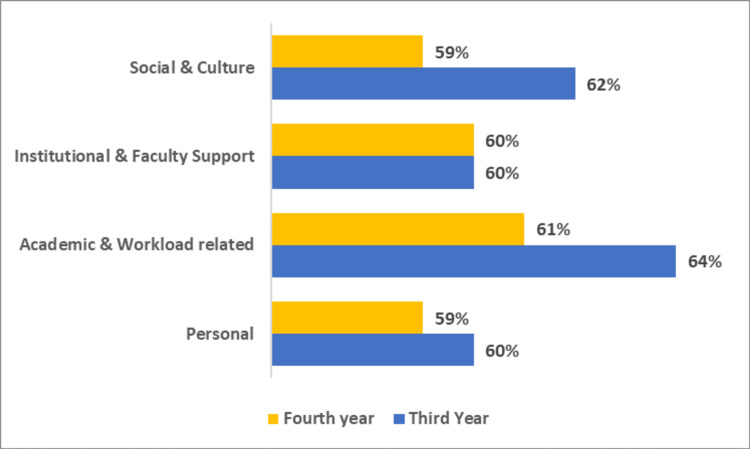
Percentage distribution of leadership challenges among senior nursing students

Figure 3 shows that third-year students reported slightly higher challenges across most areas, especially in academic and workload-related (64%) and social-cultural aspects (62%), compared to fourth-year students. Both groups faced similar challenges in institutional cum faculty support (60%) and personal aspects (60% and 59%) in the third and fourth year, respectively.

Table 2 demonstrates statistically significant differences between third- and fourth-year nursing students across people-oriented, task-oriented, and global leadership styles (p < 0.05). The effect sizes (Cohen’s d ranging from 0.35 to 0.40) indicate small-to-moderate practical differences, suggesting that while the variations are meaningful, they are not large. Third-year students scored higher across all domains, reflecting a stronger emphasis on interpersonal and task-focused leadership, whereas fourth-year students exhibited a more balanced but slightly lower profile. These findings confirm the research hypothesis (H1). There will be a significant difference between leadership styles among senior nursing students at p < 0.05.

**Table 2:** Comparison of leadership styles among senior nursing students (N = 200) *p < 0.05

Variables	Max score	Third year (n = 100)		Fourth year (n = 100)		Mean diff	Ind "t" value	p-value	Cohen d
		Mean (mean%)	SD	Mean (mean%)	SD				
Leadership styles									
People oriented	45	37.08 (82.4)	6.122	34.68 (77.06)	7.349	2.4	2.51	0.01*	0.35
Task-oriented	45	36.69 (81.53)	6.129	34.05 (75.6)	7.009	2.64	2.83	0.004*	0.40
Global leadership styles	90	73.77 (81.96)	12.05	68.73 (76.36)	14.09	5.04	2.71	0.007*	0.38

Table 3 describes the use of thematic analysis, in which recurring codes were identified, categorized, and grouped into themes and subthemes that reflect students’ experiences and perceptions of leadership, opportunities, and challenges.

**Table 3:** Themes and subthemes based on qualitative strand

Themes	Subthemes
Concept of leadership	Guiding and supporting others
	Responsibility and accountability
Leadership styles	Autocratic tendencies
	Democratic/inclusive
	Transformational/motivational
Opportunities and enablers	Student roles and positions
	Academic/clinical activities
	Faculty/mentor support
Challenges in leadership	Fear and peer judgment
	Lack of confidence
Role of education and suggestions for improvement	Adequate preparation
	Need for practical exposure

## Discussion

The present mixed-methods study assessed leadership styles, opportunities, and challenges among senior nursing students in Chennai, revealing a predominance of team or democratic leadership styles among participants. Rather than merely reflecting preference, this pattern may indicate the influence of contemporary nursing education, which increasingly emphasizes collaborative learning, patient-centered care, shared decision-making, and interprofessional teamwork. Nursing students are frequently exposed to group assignments, simulation-based training, clinical discussions, and collaborative patient management activities, all of which encourage participative leadership behaviors over authoritarian approaches. In the Indian nursing education context, where teamwork and communication are strongly emphasized during clinical postings, students may naturally adopt leadership approaches that value cooperation, mutual support, and collective responsibility. Similar findings were reported by Miles and Scott, who emphasized that participative and transformational leadership styles enhance teamwork, problem-solving, and motivation among nursing students, contributing to their readiness for future leadership roles [8].

The statistically significant differences observed between third- and fourth-year students across people-oriented, task-oriented, and global leadership domains suggest that leadership competencies evolve progressively through academic and clinical exposure. Fourth-year students demonstrated a more balanced integration of interpersonal and task-focused leadership behaviors, which may be attributed to increased clinical responsibilities, greater confidence in patient care, and repeated exposure to decision-making situations during advanced clinical training. Senior students often assume informal mentoring roles for junior students and participate more actively in ward coordination, thereby strengthening both organizational and relational leadership skills. This finding aligns with Cummings et al., who noted that experience and academic progression enhance nursing students' ability to integrate interpersonal and task-oriented leadership skills, thereby improving clinical efficiency and decision-making [9,2]. The progressive development of leadership behaviors may also reflect the impact of mentorship, faculty guidance, and experiential learning opportunities embedded within clinical education. Consistent exposure to real-world healthcare environments enables students to develop professional confidence, accountability, and adaptability, all of which contribute to leadership maturation. This interpretation is further supported by Costa et al., who highlighted that experiential learning and mentoring during clinical practice strengthen leadership competencies and professional identity in undergraduate nursing students [2,3].

The findings on leadership opportunities revealed that both third- and fourth-year students reported adequate opportunities in classroom and academic activities. Still, fourth-year students perceived greater exposure in clinical cum simulation, and extracurricular cum community domains. This trend underscores the structured integration of leadership roles into the nursing curriculum, which gradually increases students’ autonomy as they advance in their studies. Kok et al. [10] reported that leadership opportunities embedded within academic and clinical settings, such as student governance roles or simulation-based exercises, significantly enhance self-efficacy and accountability among nursing trainees. Furthermore, Alilyyani et al. [11] found that institutional support, access to leadership training, and mentorship are critical enablers of leadership development among nursing students and staff nurses, paralleling the supportive academic environment described in the current study.

In contrast, students also reported challenges in balancing academic and workload, socio-cultural expectations, and limited institutional cum faculty support for leadership initiatives. These challenges mirror the barriers described by Cummings et al., who noted that heavy academic demands, hierarchical educational structures, and insufficient mentorship often hinder leadership development among nursing undergraduates [12]. Similarly, Anunciada et al. emphasized that the organizational climate and the perceived lack of empowerment can diminish students’ motivation to assume leadership responsibilities [13]. The qualitative findings of the present study further supported these quantitative results, highlighting themes such as fear of peer judgment, lack of confidence, and inconsistent opportunities to lead, issues that are deeply rooted in social and institutional culture. Participants described the need for consistent mentorship, equitable distribution of leadership opportunities, and inclusion of practical leadership modules within the curriculum.

Moreover, the presence of transformational and democratic elements in students’ perceptions of leadership indicates a positive shift from traditional hierarchical models toward participatory and empathetic leadership practices. This evolution aligns with contemporary nursing leadership frameworks that emphasize emotional intelligence, collaboration, and shared governance [14]. Studies by Conroy et al. and Miles & Scott reaffirm that transformational leadership traits, such as inspiration, individualized consideration, and intellectual stimulation, are strongly associated with improved academic outcomes, student engagement, and future leadership potential in clinical settings [7,14].

The study’s findings also reveal the importance of early exposure to leadership roles in shaping students’ self-efficacy and career orientation. Many participants acknowledged that being assigned as class representatives, Student Nurses Association executive members, or clinical team leaders enhanced their confidence and communication skills. Costa et al. [2] argued that structured leadership exposure during undergraduate training enhances students’ readiness for complex healthcare environments by improving critical thinking, collaboration, and adaptability. Similarly, Kok et al. [10] observed that integrating leadership learning into daily academic and clinical routines fosters the emergence of reflective, competent nurse leaders capable of driving healthcare transformation.

Despite these positive trends, the current study highlights an ongoing need for formal leadership training programs in nursing education. Only 20% of students had undergone any formal leadership training, suggesting a gap between curricular intent and practical implementation. This gap supports findings from Cummings et al. and Alilyyani et al., who emphasized that leadership competencies should be systematically nurtured through workshops, simulation-based learning, and continuous mentorship rather than being left to incidental opportunities [11,12]. Furthermore, the American Association of Colleges of Nursing recognized leadership development as a critical outcome of baccalaureate education. It is recommended to incorporate structured leadership modules into nursing curricula to prepare graduates for evolving healthcare leadership roles [15].

Overall, the findings of this study underscore that leadership development among nursing students is a gradual, experience-driven process influenced by exposure, mentorship, and institutional culture. The integration of leadership education within both classroom/academic and clinical/simulation training enhances the holistic growth of future nurses, aligning with global standards for professional nursing education. Addressing the identified challenges through targeted mentorship, curricular reforms, and leadership simulation exercises can further strengthen the nursing education leadership pipeline in India.

Theme 1: concept of leadership

Students described leadership as both a guiding force and a responsibility. Participant 1 viewed leadership as “guiding my friends for the right pathway,” emphasizing the relational and ethical dimension of leadership. Participant 2 added that leadership involves accountability, stating, “Leadership is not just sitting in a big chair… but being responsible for the work and goals.” Participant 3 echoed this, saying that leadership means being entrusted with responsibilities by higher authorities. These perspectives reflect a transformational leadership orientation in which influence and mentorship are central [8]. Senior nursing students perceive leadership as a blend of guidance, responsibility, and trust. Their understanding aligns with leadership models that emphasize ethical influence and peer support.

Theme 2: leadership styles

Students identified a range of leadership styles that they have experienced in both academic and clinical settings. Participant 4 noted, “Most valued, I would say autocratic… mostly they are being so autocratic,” while Participant 5 observed that “autocracy will be there… superiors will be guiding.” These responses suggest that hierarchical leadership remains dominant. However, Participant 6 offered a balanced view: “Sometimes autocratic, sometimes democratic, both play a role,” and Participant 7 emphasized empathy, stating, “We should understand them more than give orders.” Participant 8 preferred a motivational approach, saying, “I choose the transformational style… we will motivate them to attain.” These findings are consistent with those of Cummings et al., who found that democratic and transformational styles are associated with better outcomes in nursing practice [15]. While autocratic leadership remains prevalent, students express a clear preference for democratic and transformational leadership styles that foster inclusivity and motivation.

Theme 3: opportunities and enablers

Students reported leadership opportunities through formal roles, academic tasks, and mentorship. Participant 1 shared, “In the third year, I was selected as SNA secretary… it was a great opportunity,” and Participant 2 listed multiple roles held since the first year. Participant 3 described academic leadership through practice teaching, while Participant 4 noted, “Handling 10 members itself is a task… teachers keep faith in us.” Faculty support was a recurring enabler, with Participant 2 stating, “My mentor encouraged me in many ways,” and Participant 3 adding, “Teachers built confidence… encourage students to become leaders.” These findings support AACN’s emphasis on integrating leadership development into nursing education. Leadership development is facilitated through structured roles, academic engagement, and faculty mentorship [15,2]. These enablers are essential for cultivating leadership readiness among nursing students.

Theme 4: challenges in leadership

Students expressed emotional and psychological barriers to leadership. Participant 1 admitted, “One day I thought I did not want to become a leader anymore,” reflecting self-doubt. Participant 7 shared, “Peer judgment keeps me away from being a leader,” while Participant 3 said, “I feel like I am not that consistent… maybe others can do it better.” Participant 8 added, “In every situation, I feel a lack of confidence and peer judgment.” These challenges mirror findings by Alilyyani et al., who reported similar concerns among intern nursing students. Despite exposure to leadership roles, students face internal challenges such as fear of judgment and low confidence. Addressing these through supportive environments and confidence-building strategies is critical [11].

Theme 5: role of education and suggestions for improvement

Students acknowledged the value of leadership education but emphasized the need for practical exposure. Participant 6 stated, “Surely the nursing education system is developing each student,” and Participant 1 added, “Nursing education is enriching our values.” However, Participant 4 noted, “We need it to be more practical.” Suggestions included rotational leadership roles, with Participant 1 proposing, “Every month we can choose one leader,” and Participants 3 and 7 advocating for inclusive opportunities: “Every person should be provided with the opportunity.” Participant 4 recommended exposure to hospital leadership: “We can spend time with nursing directors.” These suggestions align with those of Kok et al., who emphasize experiential learning and mentorship [10]. Students value leadership education but call for more hands-on, inclusive, and rotational opportunities. Their suggestions highlight the need for curriculum reform and experiential engagement to strengthen leadership development.

Overall, the results point to a complex, context-dependent process of leadership development among nursing students, shaped by institutional culture, mentorship, experiential learning, and academic advancement. The cross-sectional design, non-probability sampling, and single-institution setting limit the results' generalisability and causal inference, even though the mixed-methods design enhances interpretation by combining quantitative and qualitative data. As a result, conclusions should be read with caution, and generalizations beyond comparable educational situations should be avoided.

## Conclusions

According to the study, senior nursing students display developing leadership skills, primarily characterized by participatory and team-oriented approaches. Variations across academic levels indicate a steady development of leadership abilities, but these results are context-specific and should be interpreted with caution. Even while students have access to a variety of leadership opportunities, there are still clear gaps in their institutional support, emotional preparedness, and formal training. Nursing students' leadership skills may be improved by using structured leadership development programs, such as mentorship, simulation-based training, and experiential learning. It is advised that future studies employ longitudinal and multi-institutional designs to enhance generalisability and strengthen the body of evidence.
